# Temperature-Dependent Water Oxidation Kinetics: Implications and Insights

**DOI:** 10.1021/acscentsci.4c01415

**Published:** 2024-12-16

**Authors:** Tianying Liu, Pan Wang, Wei Li, David Z. Wang, Damith D. Lekamge, Boqiang Chen, Frances A. Houle, Matthias M. Waegele, Dunwei Wang

**Affiliations:** † Department of Chemistry, Merkert Chemistry Center, Boston College, Chestnut Hill, Massachusetts 02467, United States; ‡ Chemical Sciences Division, Lawrence Berkeley National Laboratory, Berkeley, California 94720, United States

## Abstract

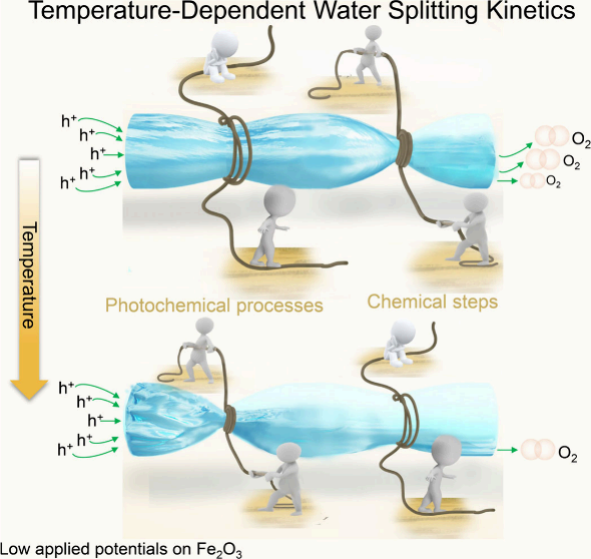

As a vital process for solar fuel synthesis, water oxidation remains a challenging reaction to perform using durable and cost-effective systems. Despite decades of intense research, our understanding of the detailed processes involved is still limited, particularly under photochemical conditions. Recent research has shown that the overall kinetics of water oxidation by a molecular dyad depends on the coordination between photocharge generation and the subsequent chemical steps. This work explores similar effects of heterogeneous solar water oxidation systems. By varying a key variable, the reaction temperature, we discovered distinctly different behaviors on two model systems, TiO_2_ and Fe_2_O_3_. TiO_2_ exhibited a monotonically increasing water oxidation performance with rising temperature across the entire applied potential range, between 0.1 and 1.5 V vs the reversible hydrogen electrode (RHE). In contrast, Fe_2_O_3_ showed increased performance with increasing temperature at high applied potentials (>1.2 V vs RHE) but decreased performance at low applied potentials (<1.2 V vs RHE). This decrease in performance with temperature on Fe_2_O_3_ was attributed to an increased level of electron–hole recombination, as confirmed by intensity-modulated photocurrent spectroscopy (IMPS). The origin of the differing temperature dependences on TiO_2_ and Fe_2_O_3_ was further ascribed to their different surface chemical kinetics. These results highlight the chemical nature of charge recombination in photoelectrochemical (PEC) systems, where surface electrons recombine with holes stored in surface chemical species. They also indicate that PEC kinetics are not constrained by a single rate-determining chemical step, highlighting the importance of an integrated approach to studying such systems. Moreover, the results suggest that for practical solar water splitting devices higher temperatures are not always beneficial for reaction rates, especially under low driving force conditions.

## Abstract 2

Varying temperature measurements reveal that the photophysical processes and the subsequent chemical steps exhibit mutual influence on each other in photoelectrochemical water oxidation reactions.

## Introduction

**Figure 1:**
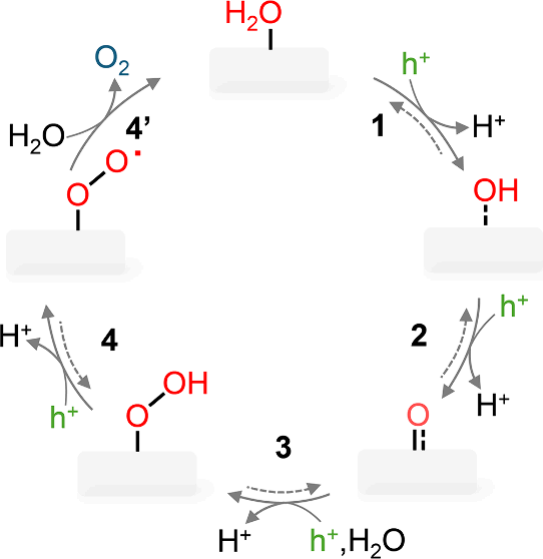
Schematic illustration of a general water oxidation catalytic cycle. A series of hole-coupled deprotonation steps occur during water oxidation, starting with the deprotonation of water to form a hydroxyl species, followed by the formation of oxo, peroxyl, and superoxo species in succession.

The water oxidation reaction is of central importance to artificial photosynthesis by providing electrons and protons that are essential for fuel-forming reactions such as hydrogen production, CO_2_ fixation, and N_2_ reduction.^1,2^ Despite decades of intense research, how to effectively carry out this reaction using inexpensive and durable systems remains a challenge. At the heart of the issue is the lack of understanding of the detailed processes that govern this reaction, especially when it is driven by photogenerated charges.^3−5^ This reaction is understood to proceed through a series of hole-capture-coupled deprotonation steps, which are schematically illustrated in Figure 1, with a total of four holes and four protons involved in a complete catalytic cycle. Under photochemical conditions, the kinetics of these chemical steps are further connected to the dynamics of photogenerated charges (including charge generation, transfer, trapping, and recombination), the details and implications of which remain under debate.^6−8^ The importance of matching the kinetics of charge generation and the subsequent catalytic steps was recently recognized by one of the coauthors (Houle) using molecular model systems.^9^ It is reasonable to expect similar effects to be applicable to other systems such as photoelectrochemical (PEC) water splitting systems. Key to the operation of a typical PEC system is the semiconductor/electrolyte interface, where a Schottky-type diode is formed due to the differences between the Fermi level of the semiconductor and the electrochemical potential of the electrolyte.^10^ Such an interface not only separates photogenerated charges but also catalyzes subsequent reactions. In the past, the two parts of this system have been often studied independently: the diode was treated as a classic Schottky-type junction to describe the photophysical behaviors of photogenerated charges, and the catalyst was treated as an electrochemical one for the understanding of the chemical steps.^11^ While convenient in borrowing knowledge from two separate fields for the study of a combined system, such a simplification misses important details concerning the synergistic effects between charge generation and catalytic processes. For instance, in studying a classical semiconductor diode with a metal charge collector, the rate of charge transfer between the semiconductor and the current collector is considered fast, and the overall charge dynamics is often considered to be limited by the photophysical processes within the semiconductor.^12^ For a PEC system, however, the charge collection proceeds through chemical reactions, which are often slow and thus limit the overall kinetics.^13^ Similarly, for a typical electrocatalytic system, only one type of charge is considered (holes for oxidation reactions); in a PEC system, the role played by complementary charges (often in the form of electron–hole recombination) is of critical importance. Taken together, we recognize the intricacies of studying integrated PEC systems for complex chemical reactions, such as water oxidation. Our goal is to fill in the knowledge gap by disentangling the synergistic effects between the photogeneration of charges and the subsequent chemical steps.

Recent research supports that surface charge accumulation is intimately connected to the chemical transformations of surface adsorbed species.^14,15^ For instance, the initial hole injection from the light absorber has been confirmed to lead to the formation of oxygen radicals on a time scale as short as picoseconds.^16^ Successive hole injection into the surface chemical species is expected to be accompanied by continued deprotonation of surface-adsorbed H_2_O species,^17^ leading to the eventual full oxidation of H_2_O and release of O_2_, as shown in Figure 1 in a simplified fashion. Assuming the photophysical processes are relatively fast, one arrives at the understanding that the overall process would be limited by the slow chemical steps.^18^ It thus follows that when the chemical steps are accelerated the rate of the overall reaction will likely increase. One parameter that is effective for speeding up chemical reactions is to raise the temperature. Although the strategy of accelerating water splitting rates by increasing the reaction temperatures has already been employed,^19−22^ systematic studies on the detailed correlation between water splitting kinetics and temperature have been scarce in the literature. We report in this work that the behaviors of a PEC system as a function of temperature can be complex, and an inverse temperature dependence was observed on hematite-based (α-Fe_2_O_3_) photoelectrodes. These results challenge the view that the overall PEC kinetics is limited by one or two chemical steps and highlight the importance of studying the system in an integrated fashion. We show in the following discussions that the photophysical behaviors of a PEC system are intertwined with the photochemical processes, highlighting that photophysical and surface chemical processes need to be treated together to understand the overall photocatalytic water oxidation. The knowledge generated by this study on the prototypical reaction of PEC water oxidation should be applicable to photoredox catalysis for chemical synthesis in general. It shows that the surface intermediates in such reactions should be considered charge-storage media, and their chemical nature and kinetics influence the photophysical processes within the semiconductor, in particular, electron–hole recombination.

## Results and Discussion

**Figure 2:**
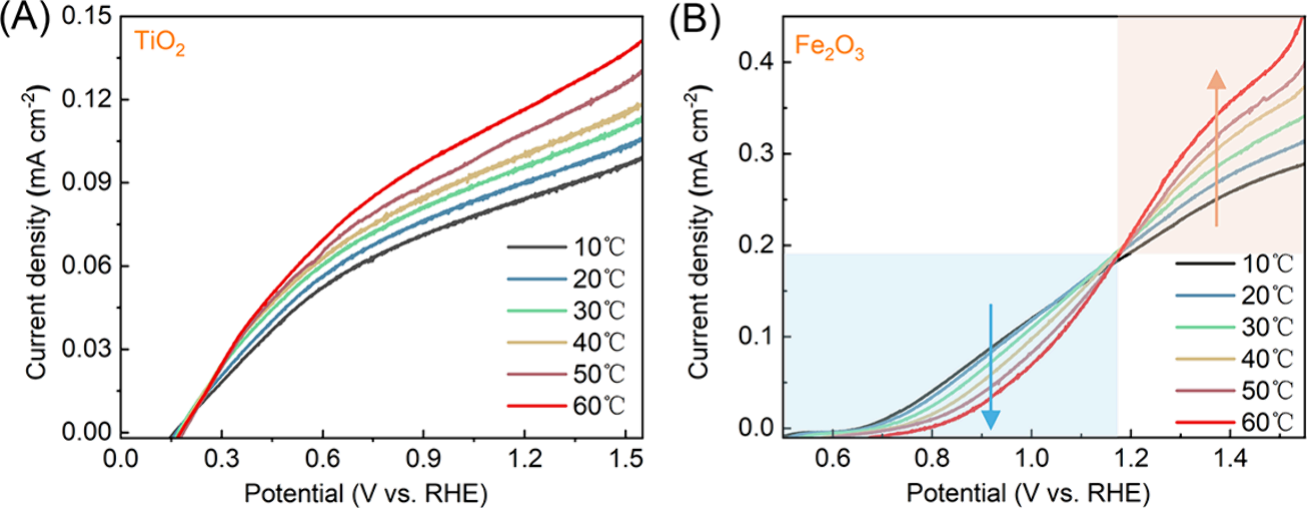
Linear sweep voltammetry (LSV) curves of (A) TiO_2_ and (B) Fe_2_O_3_ at different temperatures. The scan rate was 20 mV s^–1^. The electrodes were immersed in 1 M NaOH and illuminated with a 375 nm LED (12 mW cm^–2^) (for TiO_2_) or a 405 nm LED (33.4 mW cm^–2^) (for Fe_2_O_3_).

This body of work mainly involved two types of photoelectrodes for comparison, TiO_2_ and Fe_2_O_3_. They were prepared following procedures published in prior reports (see the Supporting Information (SI) for experimental details).^23,24^Figure 2 shows the linear sweep voltammetry (LSV) of TiO_2_ and Fe_2_O_3_ photoelectrodes immersed in 1 M NaOH at temperatures from 10 to 60 °C. As shown in Figure 2A, the water oxidation performance of TiO_2_ as measured by the photocurrent densities improved monotonically with increasing temperatures across the entire applied potential range, which was expected by assuming the chemical step(s) as limiting factors in the overall PEC water splitting.^25^ When similar measurements were carried out on Fe_2_O_3_, a more complex behavior was observed. As shown in Figure 2B, when the applied potential (*V*_app_) was >1.2 V vs the reversible hydrogen electrode (RHE, unless otherwise noted, all voltages presented hereafter are relative to this reference), the overall performance as measured by the photocurrent densities increased monotonically with increasing temperatures, similar to what was observed on TiO_2_. The result suggests that under these conditions, the overall reaction is likely limited by one or more chemical steps. At low driving forces (i.e., *V*_app_ < 1.2 *V*_RHE_), however, better water oxidation performance was observed at lower temperatures. Such inverse temperature effects for chemical reactions are rare, and there has been only one prior report that has mentioned a similar observation for PEC water splitting, to the best of our knowledge.^26^ In that work, the authors argued that the primary cause was the positive shift of the flat-band potential with increasing temperature. To test this conjecture, we performed Mott–Schottky measurements to probe the flat-band potentials (Figures S1 and S2). It was seen in Figure S3 that the difference in the flat-band potentials within the measured temperature range was negligible. Thus, we consider this explanation to be unlikely.

**Figure 3:**
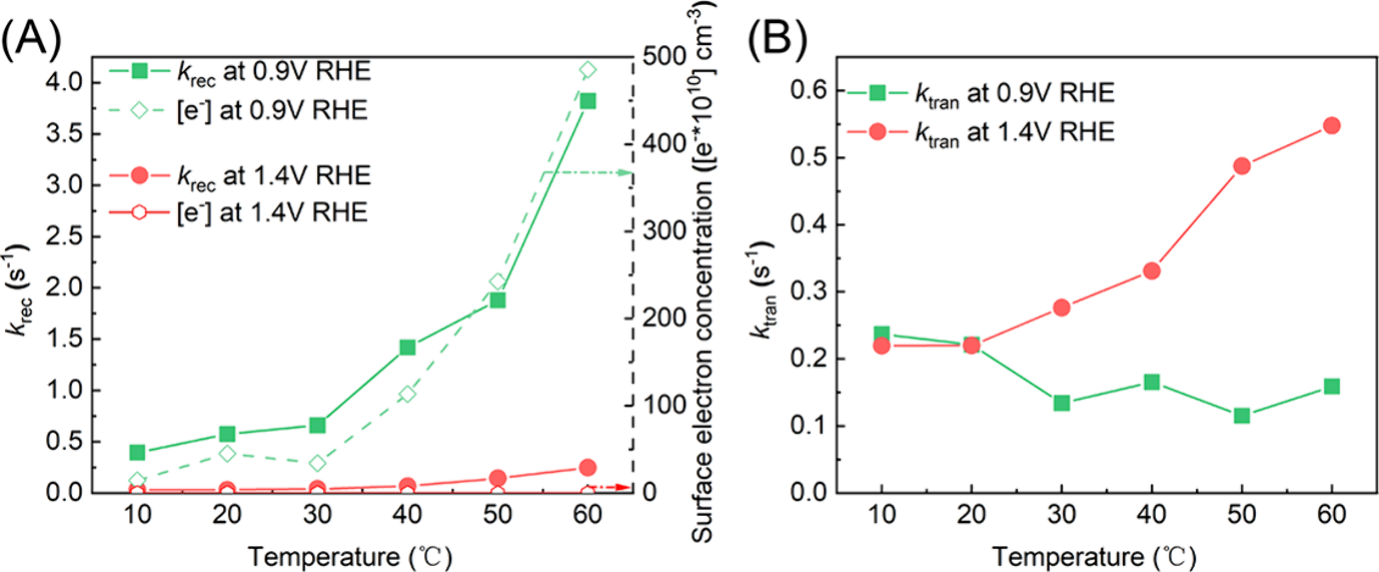
Measured (A) recombination rate constants (*k*_rec_), surface electron concentrations, and (B) charge transfer rate constants (*k*_tran_) on Fe_2_O_3_ at different temperatures.

Next, we examined another key parameter that is sensitive to temperature, the charge carrier concentration in the semiconducting photoelectrode. More specifically, we considered how the surface electron concentration changes as a function of temperature
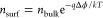
1where *n*_surf_ is the surface electron concentration, *k* is the Boltzmann constant, Δϕ is the degree of band bending, and *n*_bulk_ is the bulk electron concentration, which can be readily calculated from the Mott–Schottky measurements (Figure S3) (we caution that the exact values should be treated quasi-quantitatively as the photoanode surface deviates from a perfectly flat planar geometry, under which assumption the classical Mott–Schottky equation was derived).^27^*n*_bulk_ for Fe_2_O_3_ showed a slight increase with temperature, rising from 1.43 × 10^20^ cm^–3^ at 10 °C to 1.71 × 10^20^ cm^–3^ at 60 °C. *n*_surf_ was then obtained at varying degrees of band bending and different temperatures. At *V*_app_ = 0.9 V_RHE_ and by using the flat-band potential of 0.4 V_RHE_, we obtained an *n*_surf_ value changing from 1.44 × 10^11^ cm^–3^ at 10 °C to 4.46 × 10^12^ cm^–3^ at 60 °C, representing an increase of more than 30 times. The most direct consequence of this increase would be promoted electron–hole recombination. To test this expectation, we next conducted intensity-modulated photocurrent spectroscopy (IMPS, Figure S4) to probe the kinetic constants of the recombination process (*k*_rec_). Indeed, the *k*_rec_ increased dramatically from ca. 0.39 s^–1^ at 10 °C to ca. 3.82 s^–1^ at 60 °C (Figure 3A). By comparison, at *V*_app_ = 1.4 V_RHE_, a much greater degree of band bending (Δϕ ≈ 1 V) would be expected; as a result, *n*_surf_ was much smaller, peaking at 1.20 × 10^5^ cm^–3^ at 60 °C. Correspondingly, much lower *k*_rec_ values (ca. 0.03 s^–1^ at 10 °C and ca. 0.25 s^–1^ at 60 °C) were measured. They were significantly lower than the forward charge transfer rate constants (*k*_tran_, *vide infra*) across the entire temperature range, and their influence on the overall water oxidation performance was insignificant. Taken together, this set of data strongly supports that at low driving force with a small degree of band bending, the system is dominated by the electron–hole recombination process. The overall effect is manifested as an inverse temperature dependence of the overall reaction rates, exhibiting lower performance at elevated temperatures.

**Figure 4:**
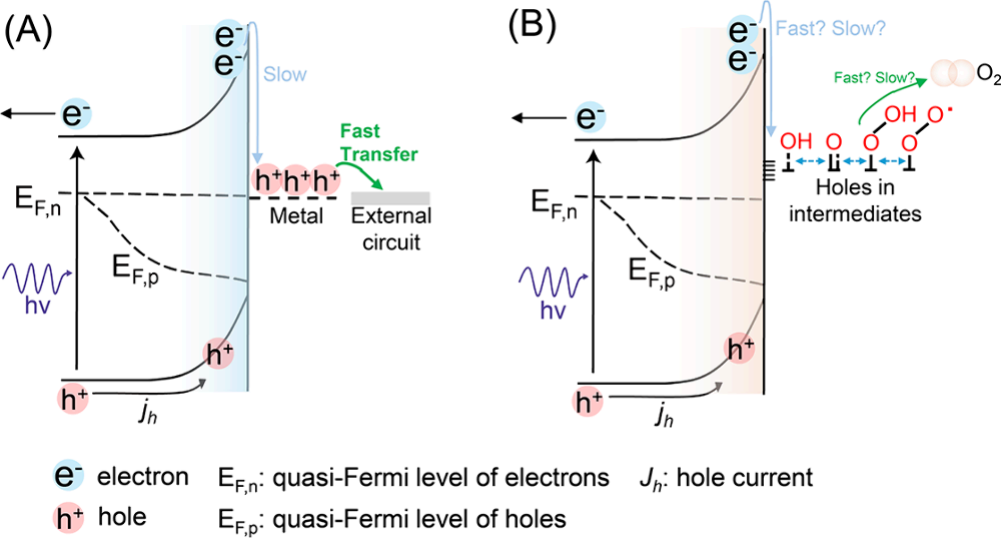
Schematic illustration of (A) the semiconductor/metal interface (SMI) and (B) the semiconductor/water interface. The key difference lies in the kinetics of charge transfer and collection, where it is fast for the former and may be slow in the latter.

When compared with Fe_2_O_3_, TiO_2_ studied here featured similar *n*_bulk_ (ca. 2.15 × 10^20^ cm^–3^); when the degree of band bending was similar (e.g., at 0.28 vs RHE, with 0.5 V of band bending), the *n*_surf_ values were similar, too (e.g., 2.66 × 10^11^ cm^–3^ at 10 °C). However, a completely different temperature dependence was observed for TiO_2_ (Figure 2A). Consistent with the LSV data, when examined by IMPS, the system appeared to be dominated by forward charge transfer with near unity charge transfer efficiency (which measures the efficiency of forward charge transfer and is obtained by *k*_tran_/(*k*_tran_ +*k*_rec_), as shown in Figure S5). Taken as a whole, this comparison raised a critical question: *What is so different between Fe*_*2*_*O*_*3*_*and TiO*_*2*_*that underpins their dramatically different charge behaviors?* This question touches on a fundamentally important consideration about the true nature of charge recombination in a PEC system. As shown in Figure 4A, in a canonical Schottky-type diode that features a classical semiconductor/metal interface (SMI),^12^ upon illumination the photogenerated minority charge would be temporarily stored in the metal contact as free charges; they would then be collected by an external circuit or annihilated by the majority charges on the surface through a surface recombination process. Key to this picture is the fast rate of charge transfer between the semiconductor and metal as well as charge collection by the external circuit, so much so that charge transfer and collection are often treated as instantaneous when describing the charge dynamics of the overall system. For most practical purposes, once separated and concentrated on the surface the photogenerated charges may be regarded as “*free*” carriers. For a PEC system, such a simplification is no longer justified. This is because various surface chemical species at different stages of chemical transformations may be involved in storing photogenerated charges. For water oxidation, these species may include hydroxyl, oxo, peroxyl, and superoxo, just to name a few (Figure 4B).^5,17^ As such, the relative rates of the chemical transformations between these species are now coupled with the photophysical behaviors of the semiconductor, creating a synergistic effect between the photoinduced charge carrier generation and the subsequent chemical catalysis.

With this insight in mind, we re-examined the model systems of TiO_2_ by computational kinetics simulations. Benefiting from the extensive literature on the various fundamental processes on TiO_2_, we performed simulations of the water oxidation kinetics on rutile TiO_2_ with a (110) exposed surface under an illumination intensity of 15 mW cm^–2^. Our results suggested that a majority of surface holes (ca. 80%) were trapped by chemical species (see Table S1). Their recombination with surface electrons will likely be manifested as the reverse of the oxidation steps. A characteristic of TiO_2_-based water oxidation is the absence of intermediates from the early steps of the catalytic cycle, with Ti-OOH^–^ or TiOOTi species as the most abundant intermediates on TiO_2_ under water splitting conditions.^28−30^ It implies that the RDS (or RDS’s) of TiO_2_-based water oxidation is likely late in the overall reaction.^30^ Consequently, for surface holes to recombine with surface electrons, it may involve the reverse reactions of multiple steps and thus features a low probability. Moreover, computational calculations suggest that TiO_2_-based water oxidation may be primarily limited by thermal steps, further supporting that electron–hole recombination is insignificant.^7^ Such an understanding is consistent with our experimental observations on TiO_2_ (Figure S5). It was revealed in recent simulation results that the chemical steps, rather than the charge transfer steps, limit the overall kinetics.^31^ Importantly, the key rate-determining step (RDS) was considered to be a late chemical step in the water oxidation cycle (for example, step 4 and step 4′ in Figure 1), either the formation of adsorbed molecular O_2_ from Ti-OOH^–^ or the desorption of molecular O_2_, consistent with literature reports as aforementioned. Consequently, the simulated water oxidation rate on TiO_2_ was found to increase linearly at elevated temperatures (Figure S6), following the same trend as observed in our experiments. This nonexponential temperature dependence behavior indicates the complexity of the multiple process-controlled reaction kinetics.

When we turn our attention back to Fe_2_O_3_, we see one apparent difference. That is, the primary intermediate of water oxidation detected on Fe_2_O_3_ (Fe=O)^32,33^ suggests the early steps of water oxidation on this material are slow, implying that intermediate species early in the water oxidation catalytic cycle are more abundant than on TiO_2_. It would involve fewer steps of the reverse reactions for holes trapped in these species to recombine with surface electrons. The net effect would be the increased probability of recombination under otherwise identical conditions. Taken together, we understand the differences in charge behaviors by TiO_2_ and Fe_2_O_3_ as a direct result of their different surface chemical kinetics. This understanding was also recognized by George et al. that the presence of different types of surface states can have an impact on the kinetics of PEC water oxidation.^34^

Our hypothesis only treats the catalytic steps in an abstract fashion; it does not necessitate the exact parallels of the detailed chemical steps on these different photoelectrode surfaces. To further reveal the difference of accumulated intermediates between TiO_2_ and Fe_2_O_3_, transient photocurrent measurements were taken on TiO_2_ and Fe_2_O_3_ at different potentials (Figure S7 and Table 2). While we caution against quantitatively comparing the absolute values of accumulated charge as different light sources were used for different materials, the data are insightful. More pronounced cathodic spikes (relative to the light-on current) were apparent on Fe_2_O_3_ than on TiO_2_. The trend suggests that Fe_2_O_3_ features more accumulated surface charges under steady-state conditions, further supporting our hypothesis.

An immediate prediction that can be derived from this hypothesis is how the forward charge transfer rate constants (*k*_tran_) change as a function of the temperature. With the assumption that increasing temperature will accelerate the chemical steps, we expect increasing *k*_tran_’s when the temperature is higher. This was indeed observed in our experiments, where at 1.4 V vs RHE, *k*_tran_ increased from ca. 0.22 s^–1^ at 10 °C to ca. 0.55 s^–1^ at 60 °C. When the surface electron concentrations are too high (i.e., at low driving forces such as *V*_app_ = 0.9 V vs RHE), however, the system would be dominated by charge recombination, and increasing the temperature creates two competing effects: on the one hand, more surface electrons are expected at high temperatures and, hence, greater electron–hole recombination, which would be detrimental to forward charge transfer; on the other hand, faster chemical steps are expected at higher temperatures, which would accelerate forward charge transfer. When combining these two competing effects, the *k*_tran_’s exhibited a slight decrease as the temperature was increased from 10 to 60 °C, as shown in Figure 3B.

**Figure 5:**
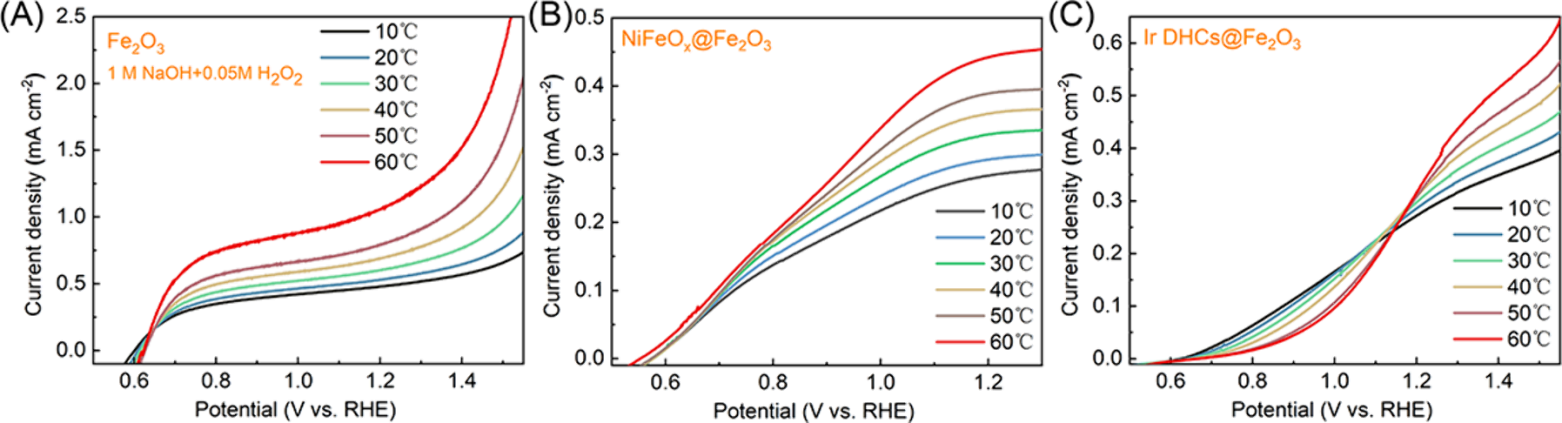
Control temperature-dependence experiments on Fe_2_O_3_ with (A) an addition of 0.05 M H_2_O_2_ in 1 M NaOH as the electrolyte, (B) NiFeO_*x*_ as the cocatalyst on Fe_2_O_3_, and (C) Ir DHCs as the cocatalyst on Fe_2_O_3_.

At the heart of the hypothesis is the understanding that the inverse temperature dependence, as shown in Figure 2, is a manifestation of the nature of surface chemical reactions. It predicts that when the surface chemistry is altered, the inverse temperature dependence may no longer be observed. To test this prediction, we added 0.05 M H_2_O_2_ as hole scavengers and performed similar varying temperature measurements.^35^ The idea was that when photogenerated holes are primarily collected by a fast reaction (H_2_O_2_ oxidation), the probability of reverse reactions and, hence, electron–hole recombination would be greatly reduced; as a result, the inverse temperature dependence would be absent. This expectation was indeed confirmed experimentally, as shown in Figure 5A. Our next control experiment involved the application of cocatalysts known for their water oxidation activities. The first cocatalyst we tried was amorphous NiFeO_*x*_ prepared by a photochemical deposition technique, which yielded a thin but complete coverage on Fe_2_O_3_.^36,37^ Now, the entire water oxidation catalytic cycle is expected to proceed on NiFeO_*x*_. A positive dependence of the overall water oxidation performance on temperature was observed, as shown in Figure 5B, suggesting that fewer intermediates early in the water oxidation catalytic cycles are available on NiFeO_*x*_. It would be interesting for future research to employ spectroscopic techniques to further probe water oxidation processes to support this understanding. Lastly, we employed Ir dinuclear heterogeneous catalysts (Ir DHCs)^38,39^ for another control experiment. Ir DHCs are unique in that they feature atomically dispersed active sites; while they are highly effective in promoting water oxidation, their surface coverage is sparse, leaving most of the Fe_2_O_3_ surface exposed to the electrolyte. Under water oxidation conditions, these exposed sites are still active toward water oxidation and will likely be occupied by various water oxidation intermediates. Their reverse reactions are therefore expected to contribute to the electron–hole recombination. The net result would be greater recombination at elevated temperatures under a low degree of band bending, which was exactly what we observed in our experiments (Figure 5C). Notably, however, the photoelectrode featuring Ir DHCs did increase the overall water oxidation performance as compared with that of bare Fe_2_O_3_ when one compares the data in Figure 5C with those in Figure 2B. These results further strengthened our earlier reports on the effects of surface anchored molecular catalysts for water oxidation, which argued that the enhancement was mainly due to increased forward charge transfer with minimum effect on recombination.^38^ The inverse temperature effect observation provides additional support.

In conclusion, a distinct temperature dependence of the water oxidation kinetics on TiO_2_ and Fe_2_O_3_ was observed. The water oxidation performance of TiO_2_ improved monotonically with increasing temperatures across the entire applied potential range. In stark contrast, the performance of Fe_2_O_3_ increased with temperature at high applied potentials (*V*_app_ > 1.2 V vs RHE) but decreased with temperature at low applied potentials (*V*_app_ < 1.2 V vs RHE). The slower kinetics at elevated temperatures on Fe_2_O_3_ at low driving force were new and may be attributed to the increased electron–hole recombination on the surface. The different temperature dependences observed for TiO_2_ and Fe_2_O_3_ were ascribed to their differing surface chemical processes: on TiO_2_, a late RDS (or RDS’s) was suggested to reduce the presence of intermediates from the early steps of the catalytic cycle, leading to a low probability of recombination with surface electrons; on Fe_2_O_3_, the slow early steps of water oxidation were thought to leave abundant intermediate species early in the water oxidation catalytic cycle, increasing the probability of recombination. This work underscores the true nature of charge recombination in a PEC system, which reflects how charges are distributed in the various steps of a complex surface chemical reaction. These results further challenge the canonical view that the photochemical and photophysical processes in a practical PEC system can be well separated. Our observations emphasize the importance of studying the system in an integrated manner. More importantly, the decreased performance of Fe_2_O_3_ at elevated temperatures indicates that higher temperature is not always beneficial for the reaction rate, especially under low driving force conditions.

## Supporting Information Available

Detailed experimental methods, computational details, Mott–Schottky plots, temperature dependence on donor density and flat band position, IMPS original data, charge behavior on TiO_2_, and computational temperature dependence water oxidation results (PDF)
