# Supplementary material for: Temperature-Dependent Water Oxidation Kinetics: Implications and Insights
Source: ACS Cent Sci. 2024 Dec 16;11(1):91–7. doi: 10.1021/acscentsci.4c01415 (PMC11758372; doi:10.1021/acscentsci.4c01415)
Supplement: Supplementary file 1 — oc4c01415_si_001.pdf [file oc4c01415_si_001.pdf]

Supporting information

Temperature-Dependent Water Oxidation Kinetics: Implications and Insights

Tianying Liu[1], Pan Wang[2], Wei Li[1], David Z. Wang[1], Damith D. Lekamge[1], Boqiang Chen[1], Frances A. Houle[2], Matthias M. Waegele[1,*], Dunwei Wang[1,*]

[1]Department of Chemistry, Merkert Chemistry Center, Boston College, Chestnut Hill, Massachusetts, 02467 United States.

[2]Chemical Sciences Division, Lawrence Berkeley National Laboratory, Berkeley, California, 94720, United States.

E-mail: waegele@bc.edu, dunwei.wang@bc.edu

Keywords: Photoelectrochemistry, hematite, TiO_2, water splitting, temperature dependence.

Experimental

1. Hematite (Fe_2O_3) synthesis

Fe_2O_3 was synthesized using an established solution synthesis method.[1] A fluorine-doped tin oxide (FTO) coated glass slide (~7 Ω sq^{-1}, Sigma) was cleaned sequentially with acetone, methanol, isopropyl alcohol, and deionized water. The clean FTO substrate was then immersed in a solution of 0.15 M $FeCl_3·6H_2O$ (97%, Alfa Aesar), 1 M $NaNO_3$ (99%, Alfa Aesar) and heated at 100°C for 1 hour. Kapton tape was applied to the backside of the glass to avoid backside deposition. After the growth process, the substrate was rinsed with deionized water and dried using a flow of nitrogen gas. The sample was then annealed when the furnace reached 800 °C for 5 min to convert the resulting FeOOH to Fe_2O_3. The process was repeated an additional time to fix surface structural defects.

2. TiO_2 synthesis

TiO_2 was deposited on the same above mentioned FTO glass by using the atomic layer deposition method.[2] Briefly, $Ti(i\text{-}PrO)_4$ (99.999% trace metals basis, Sigma-Aldrich) was heated to 75°C and used as the Ti precursor. Deionized water at room temperature was used as the oxygen precursor. The pulse and purge time for $Ti(i\text{-}PrO)_4$ and water was 0.1 s and 5 s, and 0.01 s and 10 s, respectively. The reaction was performed at 275°C with a constant flow of N_2 (UHP, 99.999%, Airgas) at 20 sccm. A 1500 cycles growth of TiO_2 was applied for this study, with an estimation thickness of ~ 48 nm by using ellipsometry.

3. Ir DHCs@Fe_2O_3 synthesis

The synthesis of the Ir homogeneous dimer complex (Ir homo-dimer) was conducted using established procedures.[3] To prepare a 1.25 mM Ir homo-dimer, 0.05 mmol of [Cp*Ir(pyalk)(OH_2)]HSO_4 was dissolved to 20.0 mL of deionized water to form a 2.5 mM solution of [Cp*Ir(pyalk)(OH_2)]HSO_4. The solution was stirred for approximately 1 min at room temperature until it became clear. Subsequently, 5.0 mmol of $NaIO_4$ (99%, Thermo Scientific Chemicals) was added, causing immediate bubbling. The mixture was stirred at room temperature in air for an additional 2 h, during which the color of the solution changed from red to green to blue. A Fe_2O_3 substrate was then immersed in the Ir homo-dimer solution for 16 h. The electrodes were then thoroughly rinsed with deionized water to form the Ir intermediate. The organic ligands of the intermediate were removed through photochemical treatments using a UVO cleaner system (Jelight Company Inc.) equipped with a UV light for 20 min.

4. $NiFeO_x$@Fe_2O_3 synthesis

Iron(III) 2-ethylhexanoate (50 % w/w in mineral spirits, Strem Chemicals) and nickel(II) 2-ethylhexanoate (78 % w/w in 2-ethylhexanoic acid, Strem Chemicals) were mixed in a 1:1 mass ratio.[4] An appropriate amount of hexane was added to this mixture to achieve a solution of the metal complex with a total concentration of 15 % (w/w). Subsequently, 5.0 μL of this solution was dropped directly onto the surface of a fabricated Fe_2O_3 electrode, with exposed electrode surface areas of around 0.050 cm^2. The thin film was dried in the air for 10 min before being irradiated with a UVO cleaner system (Jelight Company Inc.) for 3 h. The electrode with the $NiFeO_x$ coating was then annealed in an oven at 100 °C for 1 h.

5. Electrode fabrication

The prepared samples were connected with a Cu wire using Ag paste (MG Chemicals, 8331D Silver Conductive Epoxy Adhesive) and protected with non-conductive epoxy (J-B Weld 8272 MarineWeld Marine Epoxy) to leave an electrode surface area around 0.050 cm^2.

6. PEC characterizations and temperature control

PEC characterizations were carried out by using a ModuLab® XM potentiostat. A three-electrode configuration was utilized, with a Pt wire as the counter electrode, saturated calomel electrode as the reference electrode, and the fabricated electrode as the working electrode. The electrolyte was 1.0 M NaOH (≥97%, Sigma-Aldrich). For the controlled experiment, 0.050 M of H_2O_2 (30 wt % in H_2O, Sigma-Aldrich) was added to the 1.0 M NaOH solution. The light source for Fe_2O_3 and TiO_2 was a 405 nm LED (Thorlabs, 33.4 mW cm^{-2}) and a 375 nm LED (Thorlabs, 12 mW cm^{-2}), respectively, and coupled with the ModuLab® XM DSSC software. Front-side illumination was applied in all measurements. In a typical J–V plot, the voltage was swept at a rate of 20 mV s^{-1}. A customed double layer electrochemical cell with a quartz window was used for the temperature-dependent experiments. The outer layer of the cell was connected to the water bath reflux flow (Thermo Electron Digital One Circulating Bath) to control the temperature. The test was started from low temperature 10 ℃ to high temperature 60 ℃. The temperature was stabilized for at least 5 min once reaching the target temperature. The applied potential shift with temperature was corrected by using the Nernst equation.

7. Intensity modulated photocurrent spectroscopy (IMPS) test conditions

IMPS spectra were recorded by a ModuLab XM potentiostat including a frequency response analyzer (FRA). IMPS data were measured using a 10% light intensity modulation varying between 10 kHz and 0.1 Hz. Water oxidation kinetics were studied with a light centered power of 100% of LED maximum power (33.4 mW cm^{-2} for Fe_2O_3 and 12 mW cm^{-2} for TiO_2). The same three-electrode configuration and electrolyte were employed as given in the PEC characterization section. The fundamentals of IMPS model can be found in our previous report.[3]

8. Mott-Schottky plots

The Mott-Schottky plots were recorded by using the same potentiostat and set up with PEC characterization under dark conditions. An amplitude of 5 mV and frequency of 1000 Hz were applied for both TiO_2 and Fe_2O_3. The calculated values are only accurate for perfect planar geometry . As such, the data should be interpreted with caution and treated in a quasi-quantitative manner. Nevertheless, it is noted that the data provides a meaningful comparison with literature reports utilizing similar treatments.

9. Faradaic efficiency calculations

The production of oxygen was measured by a Clark electrode (Hansatech, Pentney, United Kingdom). A two-point calibration was carried out with an air-saturated solution and a Ar-saturated solution at 298 K. 2 mL of an aqueous solution containing 1 M NaOH was added to a cylindrical quartz vessel with a diameter of 1 cm. Fe_2O_3 or TiO_2 as working electrode, an Ag/AgCl reference electrode (3M NaCl, RE5B, BASi), and a graphite rod counter electrode (3 mm diameter, 99.9995% trace metals basis, Thermo Scientific Chemicals) were used. The solution was allowed to equilibrate with air under stirring (150 rpm) for 5 min. After a steady baseline was observed,

the measurement started with applied potential at 0.9 V RHE (Figure S9 (A) and (E)) or 1.4 V RHE (Figure S9 (C) and (G)) for 5 min. The faradaic efficiency (FE) of oxygen was calculated using

$$\mathrm{FE} = \frac{4 \times r_{\mathrm{O_2}}(\mathrm{nmol \cdot mL^{-1} \cdot s^{-1}}) \times V(\mathrm{mL}) \times N_{\mathrm{A}} \times e\ (\mathrm{C})}{I\ (\mathrm{mA})} \times 10^{-4} \times 100\% \quad \text{(S1)}$$

where r_{O_2} is the O_2 generation rate, V is the volume of solution, I is the current, N_A is Avogadro's number, and e is the elementary charge. r_{O_2} was obtained by fitting a linear model to the time-dependent O_2 concentration following the potential step, as shown in Figure S9 (B), (D), (F) and (H). The time interval between 400 and 550 s was analyzed because the current reached a steady-state value in this range.

10. Computational details

The reaction-diffusion mechanism was simulated using Kinetiscope,[5] an open-access stochastic chemical kinetics program. This method is particularly useful for multiscale simulations that cover a wide dynamic range in space and time, and connect molecular-level events to experimental observables.[6–8] The kinetic simulation results provide insights into the physical state and chemical populations and intermediates at all points in time, including pre-steady-state and steady state conditions. In particular, they enable a connection to be made between photophysical processes and the slower catalytic reactions. When accurate reaction steps and rate coefficients are employed, stochastic chemical kinetics simulations yield an absolute time base for direct comparison to experimental data.

The detailed mechanism used in the present work has been reported elsewhere[9] and is summarized here. Our simulated structure for crystalline rutile TiO_2 (110) consists of two parts: a surface region (1 nm) where water oxidation takes place, and a bulk light-absorbing region (40 nm). The incident wavelength of light is 375 nm with an intensity of 15 mW cm^{-2}. The reflectivity is 20%,[10–13] and absorption coefficient α is 10^4 cm^{-1}.[10,14] The Beer-Lambert law is used to calculate the rate of charge carrier photogeneration in the bulk region as a function of depth. Following photoexcitation, electrons and holes diffuse toward the surface and into the bulk crystal, or recombine. The diffusion coefficients are 1.35×10^{-3} cm^2 s^{-1} for electrons and 10^{-5} cm^2 s^{-1} for holes.[15,16] First and second order recombination kinetics are included in the bulk TiO_2, with rate coefficients of 10^3 s^{-1} for first-order steps and 3×10^5 L $mol^{-1}s^{-1}$ for second-order steps.[16] The water oxidation reaction mechanism is taken from Wang et. al.[18] In their detailed reaction scheme, water oxidation mechanism proceeds via holes oxidizing negatively charged intermediates. Reaction sites on a Ti row or a bridging O at the TiO_2 surface can both be involved. In our model we assume that surface holes diffuse freely so that the kinetics are controlled only by the instantaneous total populations of the intermediates, not their specific locations.

The study by Wang et al reports activation energies for the various reaction steps.[18] For the present study we have assumed that transition state theory applies, and have calculated rate constants over the range 10 to 60°C using the Arrhenius equation:

$$k = A\ e^{-Ea/RT} \quad \text{(S2)}$$

where A is the pre-exponential A factor, E_a is the activation energy, R is the gas constant and T is the absolute temperature. In transition state theory, A is kT/h and also scales with T. The reaction

steps, activation energies and the rate coefficients over the T range are attached at the end of this supporting information.

The outputs of the simulations are a complete history of all reactants and products as a function of time and space. For this study, we have focused on O_2 production as a function of time to compare to experiments.

We have assumed that the diffusion coefficient for holes is independent of temperature for this work. We calculated the photocurrent for two hole diffusion coefficients (10^{-5} and 10^{-7} cm^2s^{-1}) in the manuscript describing the model development (shown in Table S2 at SI of reference 9). The difference of two orders of magnitude did not yield a significant impact on the collected current, indicating that a much smaller difference due to temperature would not be important. We note that there is no information on the temperature dependence of hole diffusion in TiO_2 in the literature, to the best of our knowledge.

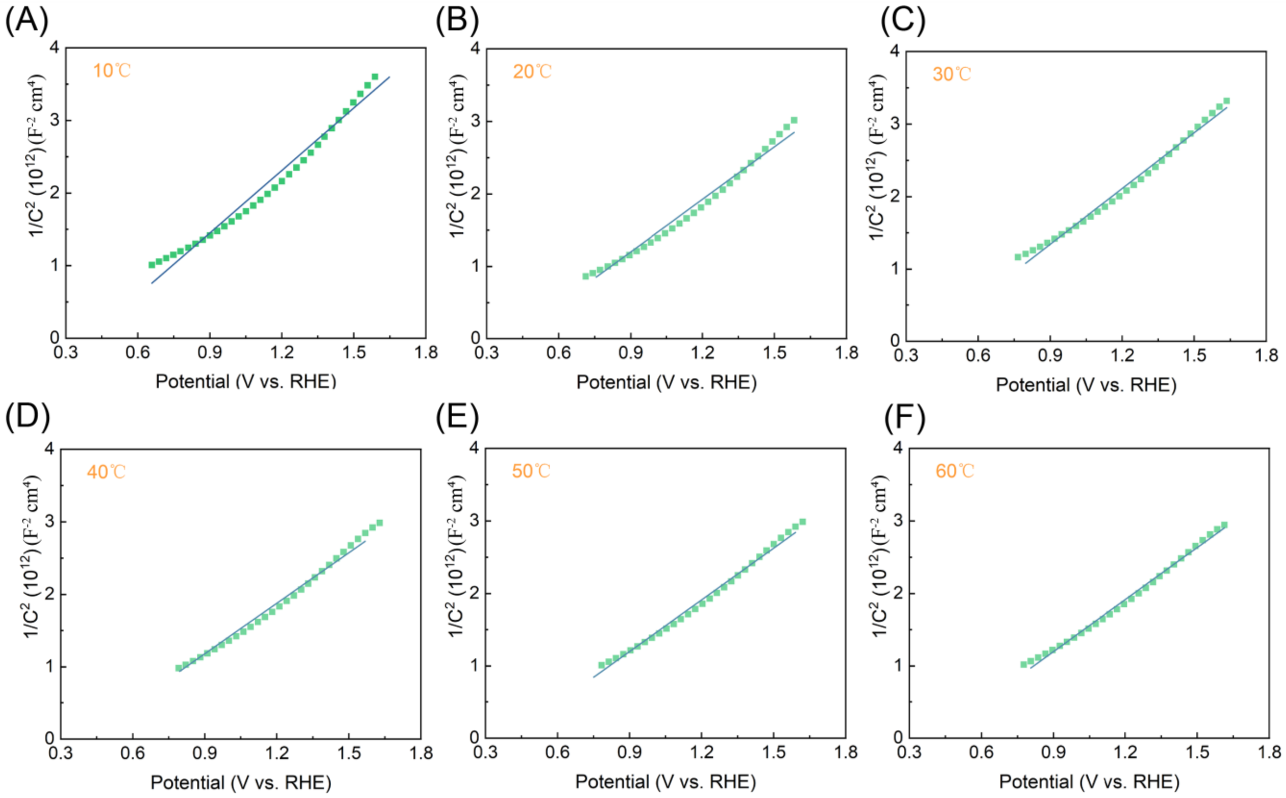

Figure S1 Mott-Schottky plots of Fe_2O_3 in the dark.

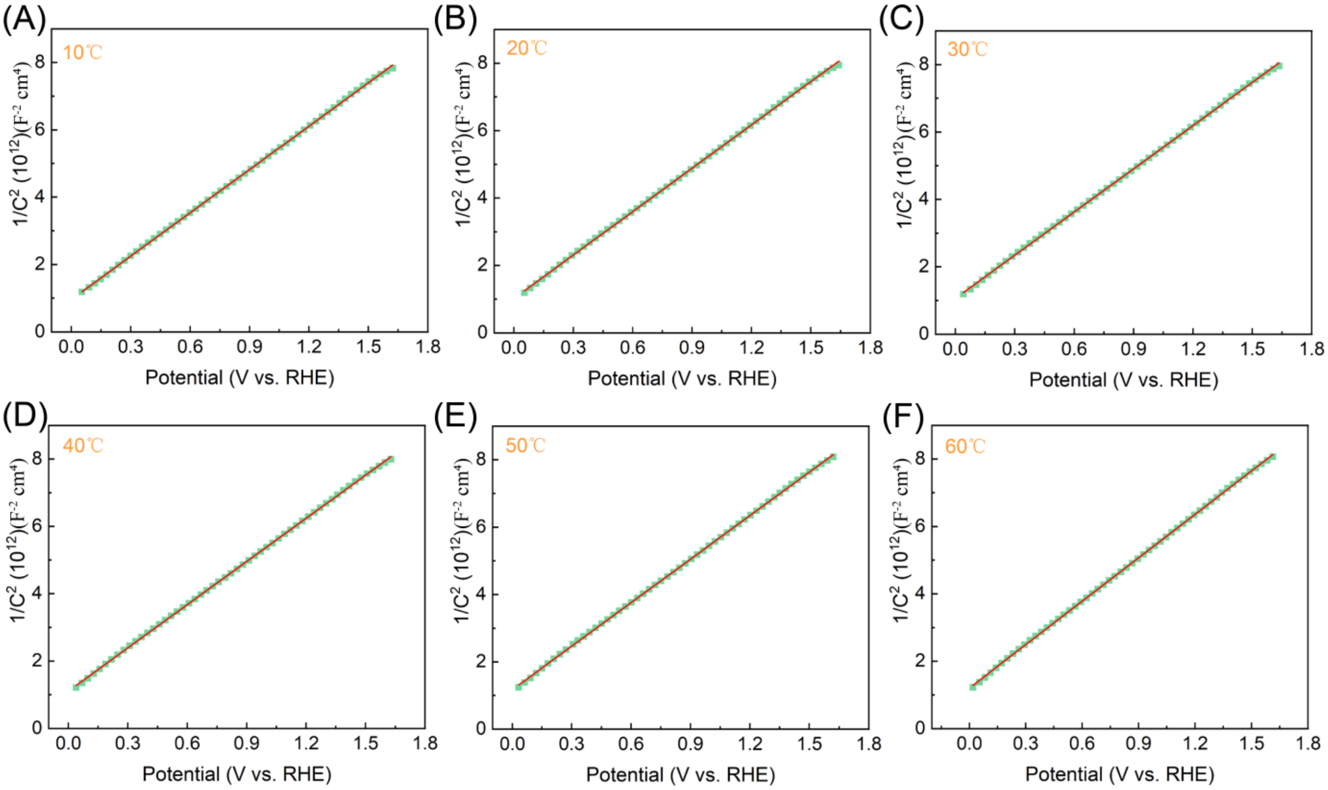

Figure S2 Mott-Schottky plots of TiO_2 in the dark.

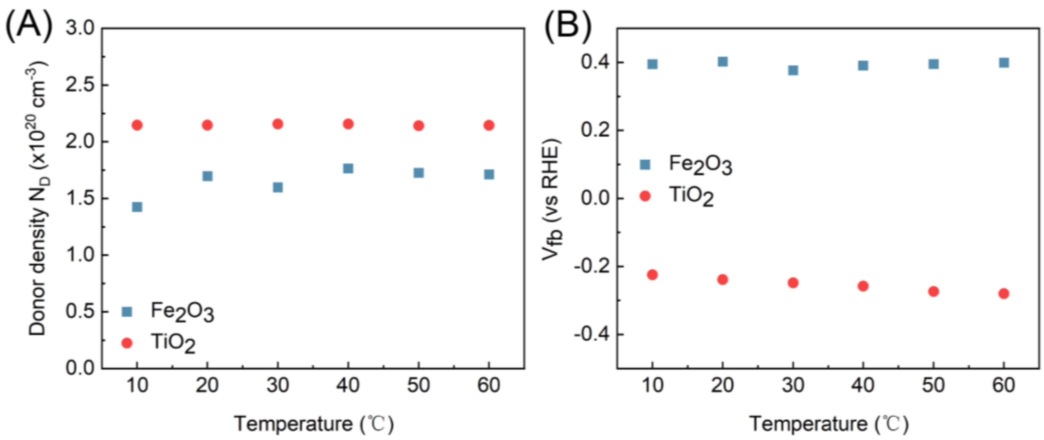

Figure S3 (A) Donor density and (B) flat band position for Fe_2O_3 and TiO_2 at various temperatures.

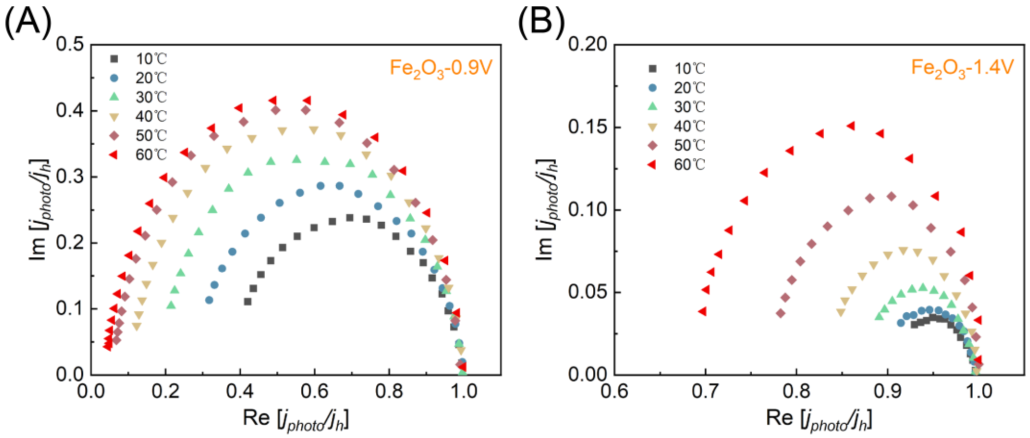

Figure S4 Normalized temperature-dependent IMPS response of Fe_2O_3 at (A) 0.9 V_{RHE} and (B) 1.4 V_{RHE}. j_h is the hole flux into the surface. j_{photo} is the photocurrent.

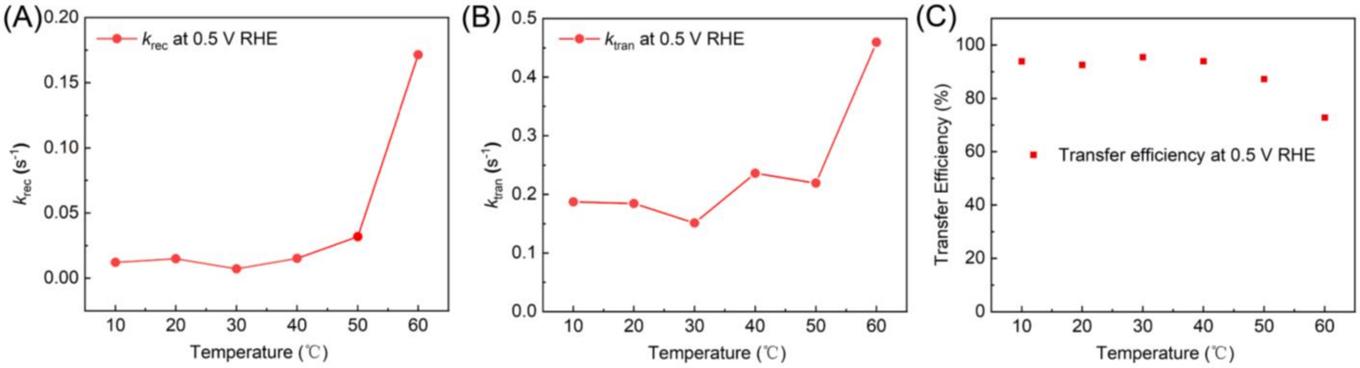

Figure S5 Measured (A) recombination rate constants (k_{rec}), (B) charge transfer rate constants (k_{tran}) on TiO_2 under different temperatures and (C) charge transfer efficiency (transfer efficiency=$k_{tran}/k_{tran}+k_{rec}$) at 0.5 V_{RHE}.

The absolute values of k_{rec} and k_{tran} between TiO_2 and Fe_2O_3 are not comparable due to the different light intensity. The near unity transfer efficiency on TiO_2 suggests that the TiO_2 system under the experimental conditions was dominated by forward charge transfer.

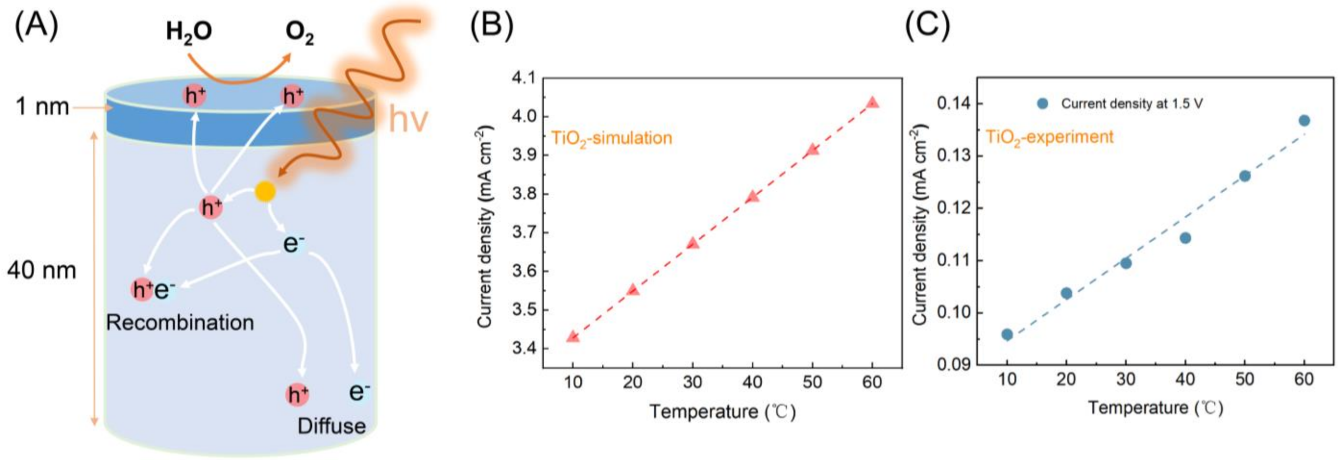

Figure S6 (A) The geometric structure of TiO_2 (110) simulated in Kinetiscope, (B) computational and (C) experimental temperature dependence of the water oxidation results on TiO_2.

There is a discrepancy in photocurrent density values between the computational and experimental results. The difference between the absolute values of the experimental and the calculated current densities is attributable to several factors, including optical losses and charge carrier losses in the film compared to the assumed perfect crystal in the computational study.

Supplementary Table 1. Detailed computational temperature dependence water oxidation results on TiO_2.

T(℃)	Surface free holes (mole/L)	Surface trapped holes (mole/L)	Bulk holes (mole/L)	Total holes (mole/L)	Photocurrent (mA cm^{-2})
**10**	9.20E-06	3.62E-05	8.75E-05	1.33E-04	3.43
**20**	9.20E-06	3.61E-05	8.75E-05	1.33E-04	3.55
**30**	9.20E-06	3.62E-05	8.75E-05	1.33E-04	3.67
**40**	9.20E-06	3.62E-05	8.75E-05	1.33E-04	3.79
**50**	9.20E-06	3.61E-05	8.74E-05	1.33E-04	3.91
**60**	9.20E-06	3.62E-05	8.75E-05	1.33E-04	4.03

Surface free holes are holes that move freely on the surface and do not participate in reactions. Surface trapped holes are holes that are trapped in active sites in the form of intermediates and participate in reactions. Bulk holes are free-moving holes in non-surface areas. In Supplementary Table 1, all values are constant except for the photocurrent, which also depends on surface reaction rates which are temperature-dependent. Because the light intensity and hole diffusion coefficient are constant in this series of temperature dependence calculations, the hole concentration does not change.

The reaction steps, activation energies and the rate coefficients over the T range

**Temperature**		10°C	20°C	30°C	40°C	50°C	60°C	
**Surface reaction (1st layer):**	**E_a (eV)**	**Rate constants**	**Rate constants**	**Rate constants**	**Rate constants**	**Rate constants**	**Rate constants**	**Unit**
Ti => $TiOH^-$ + H+sol	0.51	1.72E+06	3.63E+06	7.30E+06	1.41E+07	2.60E+07	4.65E+07	s^{-1}
$TiOH^-$ + h^+ => TiOH + h^+	0	7.00E+11	7.25E+11	7.49E+11	7.74E+11	7.99E+11	8.24E+11	L $mole^{-1}$ s^{-1}
TiOH => TiO^- + H+sol	0.41	1.87E+06	3.43E+06	6.06E+06	1.03E+07	1.71E+07	2.74E+07	s^{-1}
2 TiO^- => $TiOOTi^{2-}$	0.24	3.74E+07	5.42E+07	7.67E+07	1.06E+08	1.44E+08	1.93E+08	L $mole^{-1}$ s^{-1}
$TiOOTi^{2-}$ + h^+ => $TiOOTi^-$ + h^+	0	7.00E+11	7.25E+11	7.49E+11	7.74E+11	7.99E+11	8.24E+11	L $mole^{-1}$ s^{-1}
$TiOOTi^-$ + h^+ => O_2 + 2 Ti + h^+	0	7.00E+11	7.25E+11	7.49E+11	7.74E+11	7.99E+11	8.24E+11	L $mole^{-1}$ s^{-1}
O^{2-} + h^+ => O^- + h^+	0	7.00E+11	7.25E+11	7.49E+11	7.74E+11	7.99E+11	8.24E+11	L $mole^{-1}$ s^{-1}
O^- + TiOH => OOH^- + Ti	0.32	1.41E+06	2.28E+06	3.59E+06	5.48E+06	8.16E+06	1.19E+07	L $mole^{-1}$ s^{-1}
OOH^- + h^+ => OOH + h^+	0	7.00E+11	7.25E+11	7.49E+11	7.74E+11	7.99E+11	8.24E+11	L $mole^{-1}$ s^{-1}
OOH => OO^- + H+sol	0.23	2.99E+09	4.26E+09	5.95E+09	8.15E+09	1.09E+10	1.45E+10	s^{-1}
OO^- + h^+ => O_2 + br + h^+	0	7.00E+11	7.25E+11	7.49E+11	7.74E+11	7.99E+11	8.24E+11	L $mole^{-1}$ s^{-1}
br => H_2Obr	0	2.05E+15	2.13E+15	2.20E+15	2.27E+15	2.34E+15	2.42E+15	s^{-1}
H_2Obr => OH^-br + H+sol	0.32	7.47E+07	1.21E+08	1.90E+08	2.90E+08	4.32E+08	6.29E+08	s^{-1}
OH^-br => O^{2-} + H^+sol	0.37	9.62E+06	1.67E+07	2.80E+07	4.55E+07	7.17E+07	1.10E+08	s^{-1}

	**Recombination rate coefficients**
electron + h^+ => recom2	3.00E+05
electron + h^+ => recom1 electron trapped	1.00E+03
electron + h^+ => recom1 hole trapped	1.00E+03

**Bulk reaction (2nd-10th layer):**	**2nd-11th layers**	**Rate constants for light absorbance (mole $L^{-1}s^{-1}$)**
photon => electron + h^+ + photon	2	3.08E+00
electron + h^+ => recom2	3	2.06E+00
electron + h^+ => recom1 electron trapped	4	1.38E+00
electron + h^+ => recom1 hole trapped	5	9.28E-01
**Bulk reaction (11th layer):**	6	6.22E-01
photon => electron + h^+ + photon	7	4.17E-01
electron => gone	8	2.79E-01
h^+ => gone	9	1.87E-01
electron + h^+ => recom2	10	1.26E-01
electron + h^+ => recom1 electron trapped	11	8.41E-02

Note:

"recom2" represents the process of second-order recombination of electrons and holes.

"recom1 electron trapped" represents the process of first-order recombination of electrons being trapped.

"recom1 hole trapped" represents the process of first-order recombination of holes being trapped. "proton" represents that light produces electrons and holes. The presence of "photon" on both sides represents continuous illumination, ensuring the continuous generation of electrons and holes.

"gone" represents that electrons (holes) disappear at the bottom.

H_2O	55.4 mole/L	
hole traps	0.00415 mole/L	
Ti	0.00208 mole/L	1/2 trap sites on Ti row
O bridge	0.00208 mole/L	1/2 free sites on O bridge row
TiO_2 density	4.23000 g/ml	
TiO_2 concentration for a 1 cmx1 cm x $1e^{-9}$ cm box	52.96 mole/L	

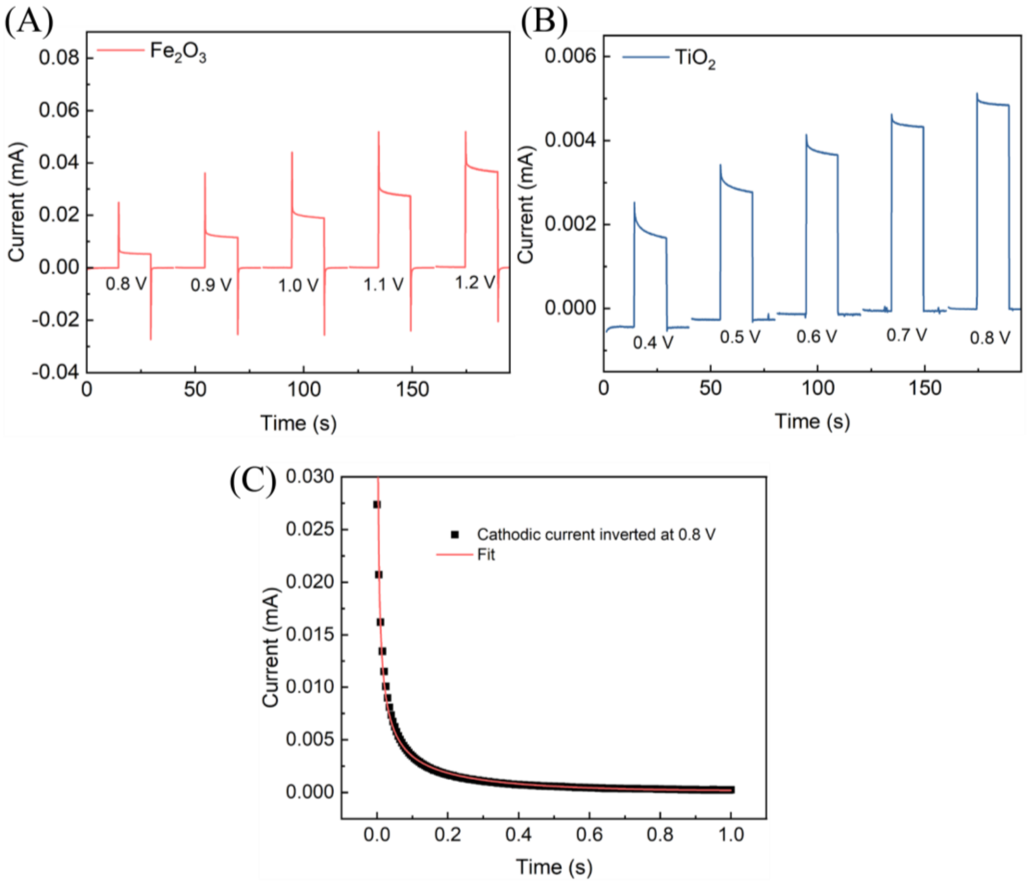

Figure S7 Transient photocurrent behavior on Fe_2O_3 (A) and TiO_2 (B) under different potentials. (C) Representative fit of equation (2) to the absolute cathodic current transient at 0.8 V_{RHE}. The fit yields the double layer charge.

Supplementary Table 2. Accumulated surface oxidative charge on Fe_2O_3 and TiO_2 and at 40°C.

Potential (V RHE)	Accumulated charge on Fe_2O_3 (C cm^{-2})	Potential (V RHE)	Accumulated charge on TiO_2 (C cm^{-2})
0.8	1.95E-5	0.4	4.96E-7
0.9	2.59E-5	0.5	1.84E-7
1	3.07E-5	0.6	1.79E-7
1.1	3.11E-5	0.7	1.86E-7
1.2	2.98E-5	0.8	1.37E-7

To obtain the total trapped charge, transient photocurrent measurements were taken at 40°C on Fe_2O_3 and TiO_2 under different potentials in 1 M NaOH. The interval between light on and off was 15 s, as shown in Figure S7. When the light was on, the photocurrents increased with the increase of the laser power. Positive and negative current spikes were observed when light was turned on and off, respectively, indicating the photoanode was charged and discharged. The transient cathodic current upon turning the light off was integrated to determine the total charge under the given potential and light intensity conditions. The first second of the absolute value of the decaying current was fit to a four-element function following Durrant et al.'s work[19,20]:

$$y = y_0 + A \cdot exp(R_0 \cdot x) + B \cdot exp(C \cdot x) + \frac{D}{\sqrt{(x)}} \quad \text{(S3)}$$

A fitting example is shown in Figure S7(C). The accumulated surface oxidative charge under the given potential was normalized by geometric surface area of Fe_2O_3 and TiO_2, as shown in Supplementary Table 2.

As different LED lights were used for TiO_2 (375 nm LED, 12 mW cm^{-2}) and Fe_2O_3 (405 nm LED, 33.4 mW cm^{-2}), the absolute values of accumulated charge amount on the different photoelectrode materials should not be compared in a quantitative fashion. The data can be qualitative compared. For instance, more pronounced cathodic spikes (relative to the light on current) were observed on Fe_2O_3 than TiO_2, indicating that Fe_2O_3 featured more accumulated surface charges under steady state conditions.

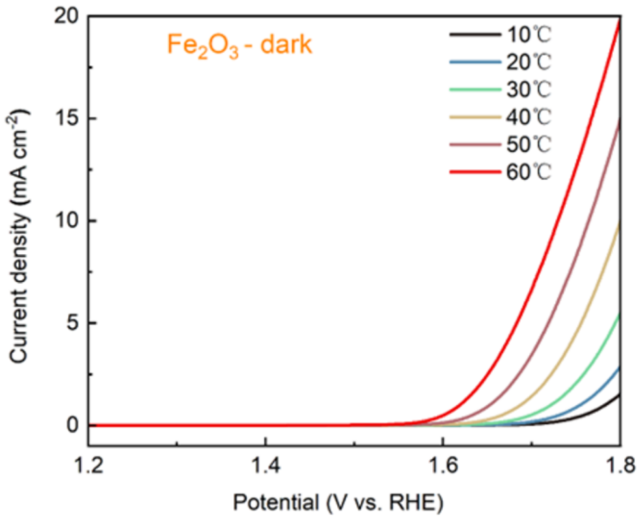

Figure S8 Temperature dependence experiments on Fe_2O_3 under dark conditions in 1M NaOH. Dark currents were collected under conditions identical to those for photoelectrochemical measurements; temperature was the only variable, and every other parameter was fixed. No currents were measurable below 1.6 V vs. RHE. A positive temperature dependence was observed.

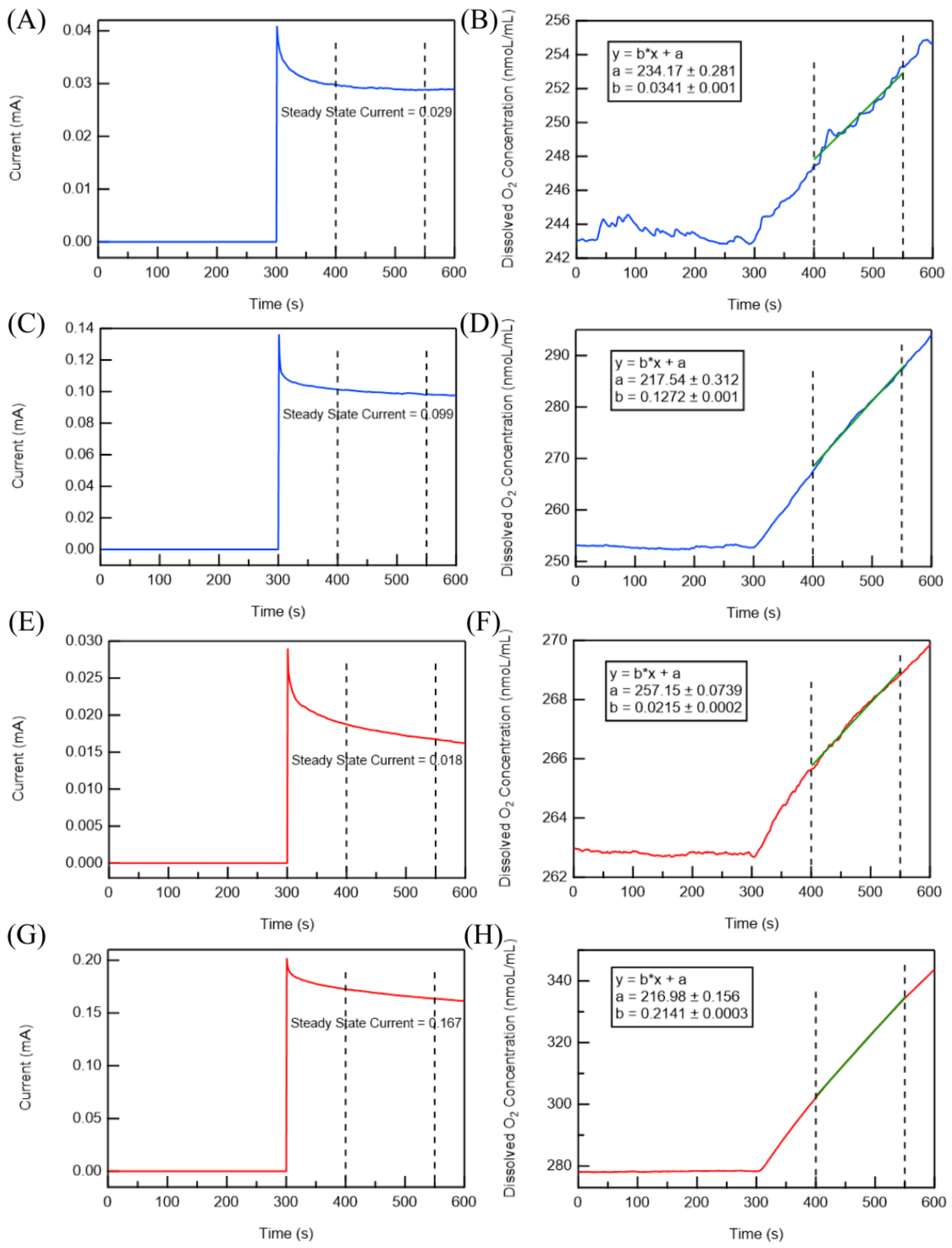

Figure S9 Photocurrent for TiO_2 at (A) 0.9 V vs. RHE, (C) 1.4 V vs. RHE, and for Fe_2O_3 at (E) 0.9 V vs. RHE, (G) 1.4 V vs. RHE. The photoelectrode was held at OCP (open circuit potential) for the first 300 s, followed by the PEC step (with light on and potential applied). O_2 evolution as detected by a Clark electrode in response to the PEC step for TiO_2 at (B) 0.9 V vs. RHE, (D) 1.4 V vs. RHE, and for Fe_2O_3 at (F) 0.9 V vs. RHE, (H) 1.4 V vs. RHE.

The calculated faradaic efficiency on TiO_2 at 0.9 V RHE and 1.4 V RHE are 90.2% and 98.8%, respectively. The calculated faradaic efficiency on Fe_2O_3 at 0.9 V RHE and 1.4 V RHE are 94.4% and

98.7%, respectively. We note that the small mismatch of faradaic efficiency is likely a result of a number of causes, including non-ideal air-tightness of the system, reduction of O_2 by the counter electrode and system errors by the detector.

REFERENCES

(1) Jang, J.-W.; Du, C.; Ye, Y.; Lin, Y.; Yao, X.; Thorne, J.; Liu, E.; McMahon, G.; Zhu, J.; Javey, A.; Guo, J.; Wang, D. Enabling Unassisted Solar Water Splitting by Iron Oxide and Silicon. *Nat. Commun.* **2015**, *6* (1), 7447.

(2) Li, W.; He, D.; Hu, G.; Li, X.; Banerjee, G.; Li, J.; Lee, S. H.; Dong, Q.; Gao, T.; Brudvig, G. W.; Waegele, M. M.; Jiang, D.; Wang, D. Selective CO Production by Photoelectrochemical Methane Oxidation on TiO_2. *ACS Cent. Sci.* **2018**, *4* (5), 631–637.

(3) Liu, T.; Li, W.; Wang, D. Z.; Luo, T.; Fei, M.; Shin, D.; Waegele, M. M.; Wang, D. Low Catalyst Loading Enhances Charge Accumulation for Photoelectrochemical Water Splitting. *Angew. Chem. Int. Ed.* **2023**, *62* (34), e202307909.

(4) Du, C.; Yang, X.; Mayer, M. T.; Hoyt, H.; Xie, J.; McMahon, G.; Bischoping, G.; Wang, D. Hematite-Based Water Splitting with Low Turn-On Voltages. *Angew. Chem. Int. Ed.* **2013**, *52* (48), 12692–12695.

(5) Hinsberg,W. D.; Houle, F. A., *Kinetiscope*, available at https://www.hinsberg.net/kinetiscope, accessed March 12, 2021, 2018.

(6) Bunker, D. L.; Garrett, B.; Kleindienst, T.; Long, G. S. Discrete Simulation Methods in Combustion Kinetics. *Combust. Flame* **1974**, *23* (3), 373–379.

(7) Gillespie, D. T. An Exact Method for Numerically Simulating the Stochastic Coalescence Process in a Cloud. *J. Atmospheric Sci.* **1975**, *32* (10), 1977–1989.

(8) MacNamara, S.; Burrage, K.; Sidje, R. B. Multiscale Modeling of Chemical Kinetics via the Master Equation. *Multiscale Model. Simul.* **2008**, *6* (4), 1146–1168.

(9) Wang, P.; Benitez, G.; Houle, F. Influence of Hole Transport and Thermal Reactions in Photo-Driven Water Oxidation Kinetics on Crystalline TiO_2. ChemRxiv 2024. DOI:10.26434/chemrxiv-2024-1xlvq.

(10) Park, Y. R.; Kim, K. J. Structural and Optical Properties of Rutile and Anatase TiO_2 Thin Films: Effects of Co Doping. *Thin Solid Films* **2005**, *484* (1), 34–38.

(11) Abdel-Aziz, M. M.; Yahia, I. S.; Wahab, L. A.; Fadel, M.; Afifi, M. A. Determination and Analysis of Dispersive Optical Constant of TiO_2 and Ti_2O_3 Thin Films. *Appl. Surf. Sci.* **2006**, *252* (23), 8163–8170.

(12) Sawicka-Chudy, P.; Sibiński, M.; Pawełek, R.; Wisz, G.; Cieniek, B.; Potera, P.; Szczepan, P.; Adamiak, S.; Cholewa, M.; Głowa, Ł. Characteristics of TiO_2, Cu_2O, and TiO_2/Cu_2O Thin Films for Application in PV Devices. *AIP Adv.* **2019**, *9* (5), 055206.

(13) Soussi, A.; Ait Hssi, A.; Boujnah, M.; Boulkadat, L.; Abouabassi, K.; Asbayou, A.; Elfanaoui, A.; Markazi, R.; Ihlal, A.; Bouabid, K. Electronic and Optical Properties of TiO_2 Thin Films: Combined Experimental and Theoretical Study. *J. Electron. Mater.* **2021**, *50* (8), 4497–4510.

(14) Kafizas, A.; Ma, Y.; Pastor, E.; Pendlebury, S. R.; Mesa, C.; Francàs, L.; Le Formal, F.; Noor, N.; Ling, M.; Sotelo-Vazquez, C.; Carmalt, C. J.; Parkin, I. P.; Durrant, J. R. Water Oxidation Kinetics of Accumulated Holes on the Surface of a TiO_2 Photoanode: A Rate Law Analysis. *ACS Catal.* **2017**, *7* (7), 4896–4903.

(15) Deskins, N. A.; Dupuis, M. Electron Transport via Polaron Hopping in Bulk TiO_2: A Density Functional Theory Characterization. *Phys. Rev. B* **2007**, *75* (19), 195212.
(16) Deskins, N. A.; Dupuis, M. Intrinsic Hole Migration Rates in TiO_2 from Density Functional Theory. *J. Phys. Chem. C* **2009**, *113* (1), 346–358.
(17) Katoh, R.; Murai, M.; Furube, A. Electron–Hole Recombination in the Bulk of a Rutile TiO_2 Single Crystal Studied by Sub-Nanosecond Transient Absorption Spectroscopy. *Chem. Phys. Lett.* **2008**, *461* (4), 238–241.
(18) Wang, D.; Sheng, T.; Chen, J.; Wang, H.-F.; Hu, P. Identifying the Key Obstacle in Photocatalytic Oxygen Evolution on Rutile TiO_2. *Nat. Catal.* **2018**, *1* (4), 291–299.
(19) Le Formal, F.; Pastor, E.; Tilley, S. D.; Mesa, C. A.; Pendlebury, S. R.; Grätzel, M.; Durrant, J. R. Rate Law Analysis of Water Oxidation on a Hematite Surface. *J. Am. Chem. Soc.* **2015**, *137* (20), 6629–6637.
(20) Righi, G.; Plescher, J.; Schmidt, F.-P.; Campen, R. K.; Fabris, S.; Knop-Gericke, A.; Schlögl, R.; Jones, T. E.; Teschner, D.; Piccinin, S. On the Origin of Multihole Oxygen Evolution in Haematite Photoanodes. *Nat. Catal.* **2022**, *5* (10), 888–899.
